# MUC16 Is Overexpressed in Idiopathic Pulmonary Fibrosis and Induces Fibrotic Responses Mediated by Transforming Growth Factor-β1 Canonical Pathway

**DOI:** 10.3390/ijms22126502

**Published:** 2021-06-17

**Authors:** Beatriz Ballester, Javier Milara, Paula Montero, Julio Cortijo

**Affiliations:** 1 Comprehensive Pneumology Center (CPC), Helmholtz Zentrum München, 85764 Munich, Germany; 2 CIBERES, Health Institute Carlos III, 46010 Valencia, Spain; julio.cortijo@uv.es; 3 Pharmacy Unit, General University Hospital, 46010 Valencia, Spain; 4 Department of Pharmacology, Faculty of Medicine, University of Valencia, 46010 Valencia, Spain; paulamonmag@gmail.com; 5 Research and Teaching Unit, University General Hospital Consortium, 46010 Valencia, Spain

**Keywords:** idiopathic pulmonary fibrosis, MUC16, transforming growth factor beta

## Abstract

Several transmembrane mucins have demonstrated that they contribute intracellularly to induce fibrotic processes. The extracellular domain of MUC16 is considered as a biomarker for disease progression and death in IPF patients. However, there is no evidence regarding the signalling capabilities of MUC16 that contribute to IPF development. Here, we demonstrate that MUC16 was overexpressed in the lung tissue of IPF patients (*n* = 20) compared with healthy subjects (*n* = 17) and localised in fibroblasts and hyperplastic alveolar type II cells. Repression of MUC16 expression by siRNA-MUC16 transfection inhibited the TGF-β1-induced fibrotic processes such as mesenchymal/ myofibroblast transformations of alveolar type II A549 cells and lung fibroblasts, as well as fibroblast proliferation. SiRNA-MUC16 transfection also decreased the TGF-β1-induced SMAD3 phosphorylation, thus inhibiting the Smad Binding Element activation. Immunoprecipitation assays and confocal immunofluorescence showed the formation of a protein complex between MUC16/p-SMAD3 in the cell membrane after TGF-β1 stimulation. This study shows that MUC16 is overexpressed in IPF and collaborates with the TGF-β1 canonical pathway to induce fibrotic processes. Therefore, direct or indirect targeting of MUC16 could be a potential drug target for human IPF.

## 1. Introduction

Idiopathic pulmonary fibrosis (IPF) is the most frequent form of interstitial lung disease with a yet unknown aetiology. IPF is mainly characterised by a progressive and irreversible injury of the lung epithelium that leads to abnormal wound healing [1,2]. The dysfunctional wound repair process secretes multiple factors that stimulate a myofibroblast overactivation and excessive extracellular matrix deposition, ultimately causing lung failure and death 3–5 years after diagnosis [3]. Myofibroblasts are characterised by secreting high amounts of a collagen-rich extracellular matrix and by expressing alpha-smooth muscle actin (α-SMA) [4,5,6]. Beyond lung resident fibroblasts, invasive myofibroblasts in IPF lungs might be originated different cell sources [7], including mesenchymal/myofibroblast transformations of alveolar type II epithelial cells (ATII) [8]. Recently, nintedanib [9] and pirfenidone [10] have been approved for the treatment of IPF. However, the positive effects are only modest and do not allow to reverse the disease course [11].

Mucins have been largely linked to IPF disease, suggesting that they act as effectors of the disease [12]. Secreted MUC5B [13] and MUC5AC [14] gene variants have been associated with IPF disease. Furthermore, we have recently reported that transmembrane MUC1 [15] and MUC4 [16] contribute intracellularly to induce fibrotic processes. Furthermore, the levels of extracellular MUC1 (KL6) have been reported markedly increased in BALF and serum from patients with various types of interstitial/fibrotic lung diseases [17], serving as a potential biomarker in IPF disease [18]. Serum levels of extracellular MUC16 (CA-125) have been also identified as a biomarker for disease progression and death in IPF patients [19,20], as well as associated with the severity and risk of cancer in ILD patients [21,22].

MUC16 is the largest of all known mucins. It consists of an extracellular N-terminal domain heavily O-glycosylated, a large tandem repeat domain interspersed with 16 SEA domains (Sperm protein, Enterokinase, and Agrin) and a C-terminal domain with several tyrosine, serine, and threonine sites for potential phosphorylation [23,24]. SEA domains have been suggested as potential carriers of the CA125 epitope [25]. Similarly to other mucins, MUC16 penultimate and final SEA domains have been proposed as putative cleavage sites [23,26]. However, potential cleavage locations as close as only 12 amino acids distal to the transmembrane domain have also been suggested to be sufficient for MUC16 cleavage and release to the extracellular space [27]. Extracellularly, CA-125 is the best-known biomarker for ovarian cancer [28], and it has been observed to be up-regulated in multiple malignancies as well as strongly associated with poor prognosis [29,30,31]. Intracellularly, a 17-kDa cleaved MUC16 C-terminal fragment that is translocated to the nucleus and binds to chromatin has been detected, suggesting involvement in the regulation of gene expression [27,32]. It is known that MUC16 participates to promote cell transformation processes, cell migration, and cell proliferation [33]. However, the functional characteristics and signalling capabilities of MUC16 that contribute to these fibrotic/metasplastic processes remain still unclear.

Transforming growth factor-beta (TGF-β) is considered to be the major cytokine involved in the excessive extracellular matrix deposition within the IPF lung [34,35,36]. TGF-β1 has been reported to lead to pulmonary fibrosis mainly through the SMAD-dependent canonical pathway [37]. However, several attempts at direct or indirect targeting of TGFβ activation have reported major side effects [38,39], due to the pleiotropic roles of TGFβ family members. We speculate that MUC16 could interact with TFGβ to promote fibrotic processes, thus hypothesising that indirect modulation of the TGFβ signal through MUC16 targeting might be an alternative IPF therapy.

In this study, we analyse the expression and distribution of MUC16 in IPF patients, as well as the interplay between TGF-β1 canonical signalling and MUC16 intracellular signalling.

## 2. Results

### 2.1. MUC16 Is Overexpressed in IPF Lung Tissue

**Figure 1:**
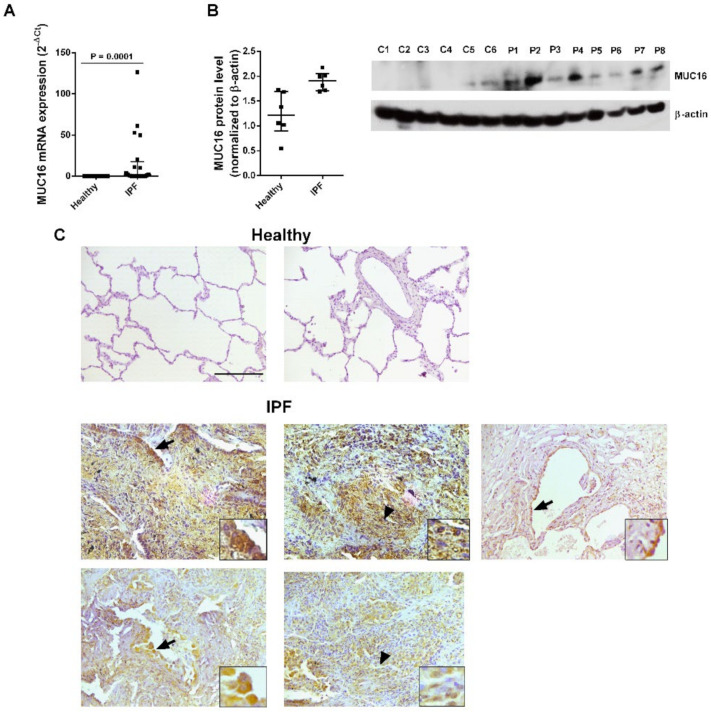
**MUC16 is overexpressed in lung tissue from idiopathic pulmonary fibrosis (IPF) patients.** Lung tissue was obtained from healthy controls (*n* = 17) and IPF patients (*n* = 20). (**A**) MUC16 mRNA expression was analysed by real-time polymerase chain reaction (PCR). (**B**) MUC16 protein expression levels were analysed by Western blotting (*n* = 14). (**C**) Immunohistochemistry of MUC16. Top panel: healthy lung sections, bottom panel: IPF lung sections. Scale bar: 100 μm. ATII cells (arrows) and fibroblasts (arrowhead) show positivity for MUC16 immunostaining. Data are shown as the ratio compared to β-actin for protein expression and 2^−ΔCt^ for mRNA levels. Data are presented as a scatter dot plot with median and interquartile ranges. *p* values are based on the Mann–Whitney test.

MUC16 transcript and MUC16 protein expression levels were higher in lung tissue from IPF patients compared to healthy subjects (Figure 1A,B). Immunohistochemical stainings of MUC16 in lung tissue from IPF patients showed MUC16^+^ staining in aberrant, hyperplastic alveolar epithelial cells (Figure 1C, arrows) and fibroblasts (Figure 1C, arrowheads) in IPF sections, and there was no or only rare MUC16 staining in lung sections from healthy subject specimens (Figure 1C). The IPF phenotype for the lungs used was further confirmed by Masson’s trichrome staining, highlighting collagen deposition in the fibrotic lungs (Supplementary Figure S1). Together, these data show that MUC16 overexpression in IPF is localised to the main cells, contributing to lung fibrosis.

### 2.2. MUC16 Collaborates with TGF-β1 to Promote the Alveolar Type II to Mesenchymal and Fibroblast to Myofibroblast Transitions

**Figure 2:**
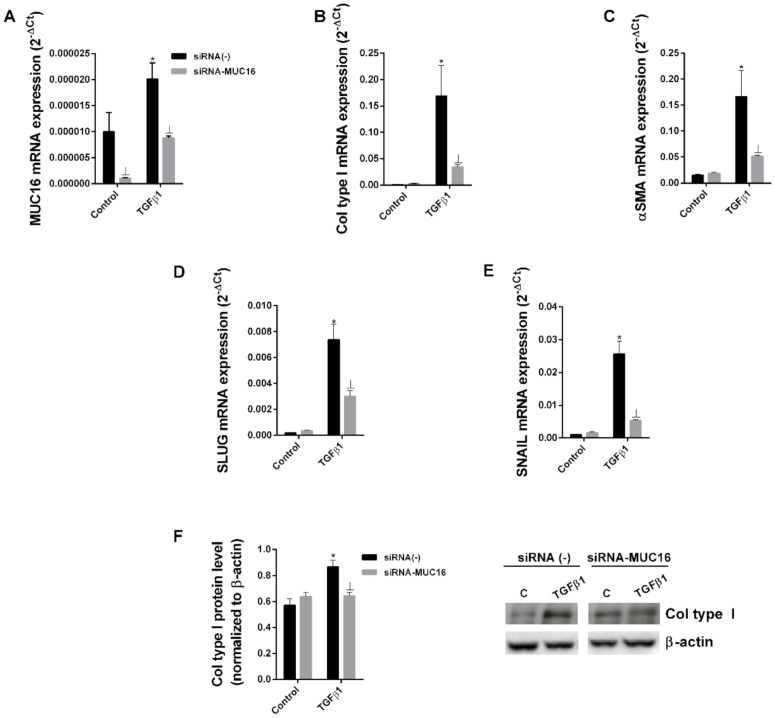
**TGF-β1 and MUC16 collaborate to induce the alveolar epithelial to mesenchymal transition.** The A549 cell line transfected with control siRNA(−) or siRNA-MUC16 was stimulated for 48 h (**A**–**E**) or 72 h (**F**) with 5 ng/mL TGF-β1 to measure MUC16 (**A**), collagen type I (**B**), α-SMA (**C**), Slug (**D**), and Snail (**E**) mRNA expression by real-time PCR and collagen type I protein levels by Western blotting, quantification was performed by densitometry (**F**). Data are expressed as 2^−ΔCt^ for mRNA levels and relative to β-actin for protein levels. The results are expressed as means ± SE. Student t-test of three independent experiments performed in triplicate. * *p* < 0.05 vs. control; Ʇ *p* < 0.05 vs. siRNA(−).

**Figure 3:**
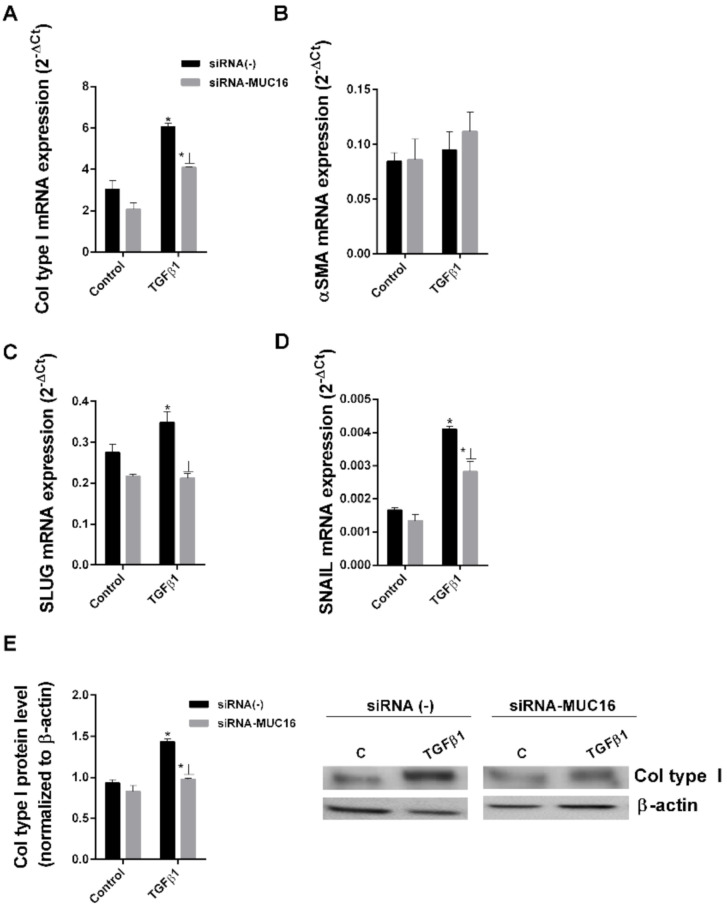
**TGF-β1 and MUC16 collaborate to induce the fibroblast to myofibroblast transition.** The MRC5 cell line transfected with control siRNA(−) or siRNA-MUC16 was stimulated for 48 h (**A**–**D**) or 72 h (**E**) with 5 ng/mL TGF-β1 to measure collagen type I (**A**), α-SMA (**B**), Slug (**C**), and Snail (**D**) mRNA expression by real-time PCR and collagen type I protein levels by Western blotting, quantification was performed by densitometry (**E**). Data are expressed as 2^−ΔCt^ for mRNA levels and relative to β-actin for protein levels. The results are expressed as means ± SE. Student t-test of three independent experiments performed in triplicate. * *p* < 0.05 vs. control; Ʇ *p* < 0.05 vs. siRNA(−) siRNA(−) + TGF-β1.

We and others have previously reported that TGF-β is able to induce EMT/FMT fibrotic processes [40,41,42]. Thus, we examined potential collaboration between TGF-β1 and MUC16 to induce EMT and FMT processes. We performed siRNA-MUC16 transfection in the bronchoalveolar cell line A549 and MRC5 lung fibroblast cell lines. siRNA transfection efficiency was confirmed (Figure 2A). TGF-β1 promoted the epithelial to mesenchymal transition in A549 cells (Figure 2), increasing the protein and/or gene expression of the mesenchymal markers, collagen type I, αSMA, SLUG and SNAIL after 48 h (Figure 2B–E) or 72 h (Figure 2F) of stimulation. However, in A549 cells transiently transfected with siRNA-MUC6, TGFβ1 did not promote the epithelial to mesenchymal transition, reducing the enhanced expression of collagen type I, αSMA SLUG and SNAIL (Figure 2). Similar results were observed in the MRC5 lung fibroblast cell line. TGF-β1 induced the fibroblast to myofibroblast transition in MRC5 cells (Figure 3), but TGFβ1 was not able to induce the FMT transition in MRC5 cells transiently transfected with siRNA-MUC16, thus reducing the increase of myofibroblast markers collagen type I, αSMA, SLUG, and SNAIL expression (Figure 3).

### 2.3. MUC16 Mediates TGF-β1-Induced Lung Fibroblast Proliferation

**Figure 4:**
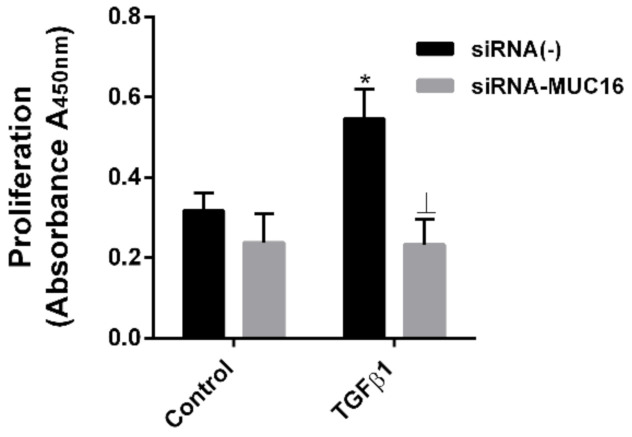
**MUC16 mediates TGF-β1-induced lung fibroblast proliferation.** TGF-β1 10 ng/mL was added in siRNA-MUC16 or siRNA(-) lung MRC5 fibroblasts during 48 h, and proliferation was evaluated by the BrdU assay. The results are expressed as means ± SE. Student t-test of three independent experiments performed in triplicate. * *p* < 0.05 vs. control; Ʇ *p* < 0.05 vs. siRNA(−) + TGF-β1.

IPF fibroblastic foci are characterised by highly TGF-β-induced active and proliferative fibroblasts [43]. We aimed to further investigate the role of MUC16 in TGF-β1-induced fibroblast proliferation. First, TGF-β1-induced proliferation was confirmed by TGF-β1 treatment at a dose of 10 ng/mL during 48 h in MRC5 fibroblasts (Figure 4). In contrast, the proliferation rate was unchanged after TGF-β1 treatment in MRC5 cells transiently transfected with siRNA-MUC16 (Figure 4).

MUC16 mediates the TGF-β1-induced canonical SMAD3 phosphorylation independently of TGF-β1-induced Wnt canonical pathway activation.

**Figure 5:**
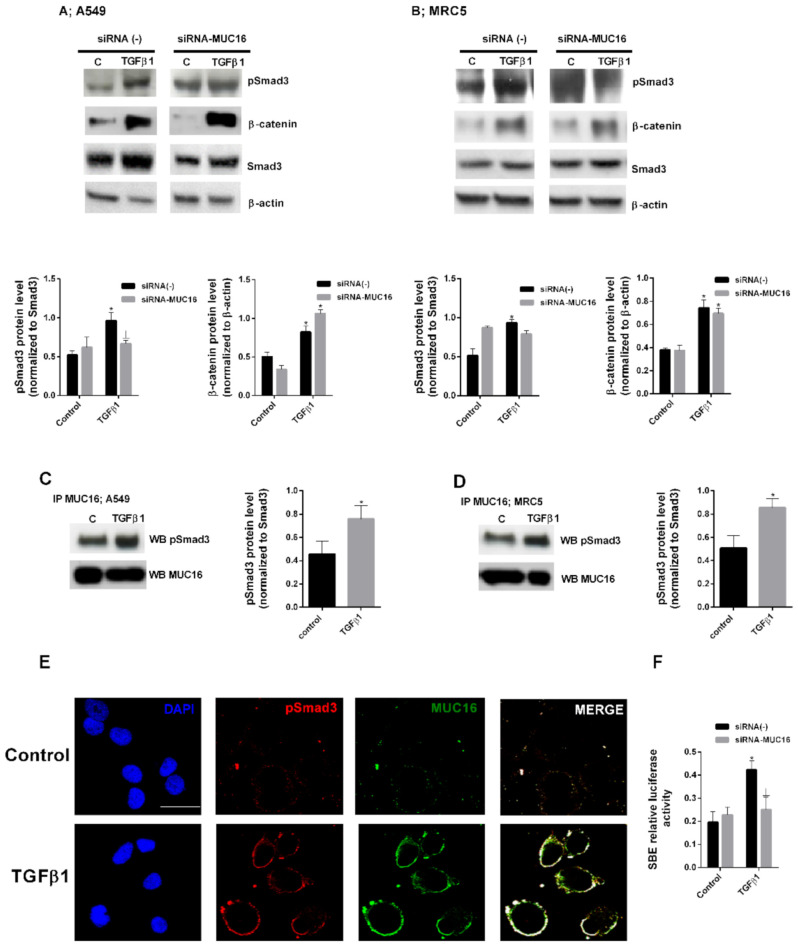
**MUC16 modulates the effect of TGF-β1 on SMAD3 phosphorylation.** (**A**) A549 and (**B**) MRC5 cell lines transfected with control siRNA(−) or siRNA-MUC16 were stimulated for 40 min with TGF-β1 10 ng/mL to measure p-Smad3 and for 48 h with TGF-β1 5 ng/mL to measure β-catenin by Western blot. A549 (**C**) and MRC5 (**D**) cells were stimulated with 10 ng/mL TGF-β1 for 40 min. Total protein was extracted and immunoprecipitated using MUC16 antibody and probed against p-Smad3 and MUC16 by Western blot (representative images are shown). (**E**) A549 cells were stimulated with 10 ng/mL TGF-β1 for 1 h. MUC16 and p-Smad3 co-localisation was analysed by confocal microscopy to generate a two-dimensional cytofluorogram that selected common localised points of both antibodies (white colour). Scale bars: 10 μm. (**F**) Smad Binding Element (SBE) measure following 10 ng/mL TGF-β1 stimulation during 18 h in A549 cells transfected with siRNA(−) or siRNA-MUC16. Data are expressed relative to Smad3 or β-actin protein level (A-D). The results are expressed as means ± SE of three independent experiments performed in triplicate. Student t-test was used. * *p* < 0.05 vs. control; Ʇ *p* < 0.05 vs. siRNA(−) + TGF-β1.

TGF-β1 induced the phosphorylation of SMAD3 in A549 and MRC5 cells (Figure 5A,B). However, the phosphorylation of SMAD3 induced by TGF-β1 was inhibited when the cells were transiently transfected with siRNA-MUC16 (Figure 5A,B). After SMAD3 phosphorylation, the nuclear transcriptional Smad-Binding Element (SBE) was activated. Here, we observed that TGF-β1-induced SBE activation was attenuated in A549 cells transiently transfected with siRNA-MUC16 (Figure 5F). By contrast, TGF-β1-induced β-catenin overexpression was not repressed by siRNA-MUC16 transfection in A549 and MRC5 cells (Figure 5A,B).

Immunoprecipitation assays revealed MUC16/p-SMAD3 protein complex formation after TGF-β1 stimulation in A549 and MRC5 cells (Figure 5C,D). Confocal immunofluorescence also demonstrated MUC16/p-SMAD3 co-localisation between following TGF-β1 stimulation in A549 cells (Figure 5E).

## 3. Discussion

The present study provides first-time evidence of MUC16 intracellular participation in IPF, which might help to understand IPF pathogenesis, as well as to identify potential drug targets.

MUC16 promotes cell transformation processes, cell migration, and cell proliferation [33]. Indeed, CA125 (MUC16) is currently a well-established serum tumour marker for ovarian epithelial cancer and may diagnose early stage cancer [44]. Antibodies such as Oregovomab and Abagovomab against CA125 have been used in clinical trials for ovarian cancer patients. However, positive outcomes have not been reported yet [45,46,47]. CA125 have been also identified as a biomarker for disease progression and death in IPF patients [19,20]. Nevertheless, CA125 alone is considered as insufficient for predicting the severity of IPF [20].

MUC16 is a transmembrane mucin that consists of three major domains: an extracellular N-terminal domain, a large tandem repeat domain interspersed with 16 SEA domains, and a C-terminal domain of 32 amino acids [23]. The N-terminal region has multiple sites for O-glycosylation, which may allow for extracellular matrix interactions. However, the cytoplasmic tail contains one serine, two threonine, and three tyrosine amino acid residues that might be potential phosphorylation sites [24]. The autoproteolytic cleavage in the penultimate and final SEA domains have been proposed as the major proteolytic mechanism of MUC16 cleavage [23,26]. Furthermore, fibrotic factors such as matrix metalloproteinase 7 (MMP-7) and neutrophil elastase are implicated in the enhanced shedding of MUC16 from the the cell surface [48,49]. It has also been suggested that potential cleavage locations as close as only 12 amino acids distal to the transmembrane domain are sufficient for MUC16 cleavage [27]. After cleavage, MUC16 C-terminal domain has been reported to translocate to the nucleus and binds to chromatin, thus participating in the regulation of gene expression [50]. The MUC16 C-terminal domain has been demonstrated to be responsible for promoting MUC16-mediated oncogenic signalling [51]. Interestingly, oncogenic signalling requires MUC16 surface interaction with Galectin-3 [52], which is a recognised therapeutic target in IPF [15,53].

Here, we hypothesised that MUC16 C-terminal domain may collaborate to induce fibro-proliferative disorders such as pulmonary fibrosis. We aimed to focus on MUC16 C-terminal subunit in both IPF tissue/cell distribution and cell function. However, the lack of commercially available antibodies targeting MUC16-C terminal domain is a potential limitation of our study.

MUC16 has been observed to be predominately expressed by goblet cells in the surface epithelium suggesting a protecting role during initial contact [54,55]. However, CA-125 was previously shown to be increased and secreted throughout the metaplastic epithelium in fibrotic lesions [19]. In this study, we confirmed MUC16 overexpression in fibrotic lung tissue. Furthermore, MUC16 cellular distribution in IPF lung tissue was confirmed to be located not only in pathologic hyperplastic alveolar type II cells but also in lung fibroblast from fibrotic foci.

TGF-β1 is defined as the main pro-fibrotic factor. However, the efforts to target TGFβ activation have major undesired effects [38,39], and clinical use has been rejected. We hypothesised MUC16 interaction with the TGF-β1 pathway to modulate key fibrotic cellular processes such as epithelial to mesenchymal transition, fibroblast to myofibroblast transition, and fibroblast proliferation [8,56]. IPF is characterised by an excessive number of persistently activated myofibroblasts [6]. Myofibroblasts are characterised by the deposition of extracellular matrix components (a characteristic shared with fibroblasts) and by the formation of the contractile apparatus (a characteristic shared with airway smooth muscle cells), that allow the myofibroblast to contract and migrate to invade lung tissue [57]. It has been suggested that myofibroblasts have multiple origins, including resident lung fibroblasts [58] and epithelial cells [59,60]. Here, we observed that the TGF-β1-induced alveolar epithelial cell to mesenchymal transition and fibroblast to myofibroblast transition were inhibited when the expression of MUC16 was repressed by siRNA-MUC16 cell transfection. However, a further Western blot analysis of TGF-β1-induced EMT and FMT markers when MUC16 is expressed/repressed should be performed to fully confirm this finding. Nevertheless, we have previously observed a high correlation between the changes detected in mRNA and protein levels after siRNA transfection of A549 and MRC5 cells [15,42]. In addition to lung fibroblasts and epithelial cells, pleural mesothelial cells are also plausible candidates of myofibroblast transformation in IPF [61,62,63,64,65]. MUC16 has been reported to bind to mesothelin, thus enhancing the epithelial mesothelial to mesenchymal transition [66]. Therefore, the role of overexpressed MUC16 on cellular transformations might have translational value to IPF. In addition to cell transformations, the fibroblast proliferative phenotype is also a key pathologic phenotype in IPF. Here, we observed that the inhibition of MUC16 using siRNA-MUC16 blocked the lung fibroblast proliferation induced by TGF-β1. The senescence phenotype of ATII and the lung fibroblast are also abundant in the lungs of IPF patients [67]. Recent studies have reported that MUC16 expression correlates with the expression of the senescence marker p16 in IPF lung tissue [68]. Therefore, we speculate that MUC16 may also collaborate with TGF-β1 to induce cell senescence.

A549 and MRC5 cell lines have been broadly used in IPF studies [15,69,70,71]. However, the fact that we used A549 and MRC5 cell lines instead of primary cells from IPF patients to explore the collaboration of MUC16 on the fibrotic effects of TGF-β1 could be interpreted as a limitation of this study. Nevertheless, the high cell number and cell stability required to perform siRNA transfection justify the use of cell lines instead of primary cells.

**Figure 6:**
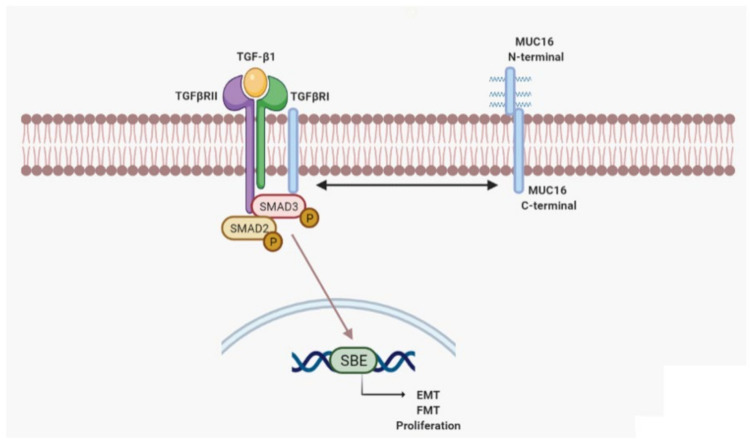
**Schematic illustration showing the collaboration of MUC16 with the TGF-β1 cellular pathway in idiopathic pulmonary fibrosis.** After TGF-β1 stimulation, MUC16 forms a protein complex with pSmad3, allowing the proper phosphorylation and the consequent activation of the Smad Binding Element (SBE) to promote the expression of pro-fibrotic markers, cellular transformations such as epithelial to mesenchymal transition (EMT), fibroblast to mesenchymal transition (FMT), and cell proliferation.

The major signalling pathway of TGF-β1 is through its canonical pathway, thus phosphorylating/activating the cytoplasmic SMAD2 and 3, which acts as a transcriptional factors to regulate proliferation and fibrotic genes. We observed that TGF-β1-induced SMAD3 phosphorylation was inhibited in alveolar epithelial cells and lung fibroblasts transiently transfected with siRNA-MUC16. These results indicate an interaction between the TGF-β1 system and MUC16. Immunoprecipitation experiments showed that TGF-β1 induces a protein complex formation between MUC16/p-SMAD3, which is co-localised in the plasmatic membrane of alveolar epithelial cells. In the cell nucleus, phosphorylated/active SMAD3 is able to activate the Smad Biding Element (SBE), which regulates the gene expression of SMAD3 pro-fibrotic dependent genes. However, when the expression of MUC16 was repressed by siRNA-MUC16 cell transfection, TGF-β1-induced SBE activation was repressed (Figure 6). Therefore, we speculate that MUC16/p-SMAD3 complex formation is required for TGF-β1 canonical signalling activation. TGF-β signalling has also been reported to regulate the canonical Wnt pathway, inducing nuclear accumulation of β-catenin [72]. Here, we observed that TGF-β1-induced β-catenin overexpression is not repressed when MUC16 is absent. However, it has been reported in the literature that the MUC16 C-terminal domain interacts with β-catenin to activate the Wnt/β-catenin signalling pathway by facilitating nuclear translocation of β-catenin. Since we assessed the expression of total β-catenin, we hypothesise that MUC16 might be no required for TGF-β1-induced β-catenin overexpression, but it is required for the transportation to the nucleus and activation of β-catenin.

In summary, the data presented in this work show MUC16 overexpression and distribution in lung tissue from IPF patients. We observed that overexpressed MUC16 cooperates with p-SMAD3 to promote fibrotic cellular disorders, thus identifying potential new targets to treat this devastating disease.

## 4. Materials and Methods

### 4.1. Patients

Human lung tissue was obtained from two types of patients (Thoracic Surgery and Pathology Services of the University General Consortium Hospital (CHGUV): (A) patients with IPF who were underwent surgery for an organ transplantation program (*n* = 20), (B) lung explant control samples from an organ transplant program in donors with normal lung function, which were not used for transplant purposes (represents lung tissue without IPF and were used as controls), that were without any lung disease (*n* = 17)).

IPF was diagnosed according to the American Thoracic Society/European Respiratory Society (ATS/ERS) consensus criteria [73]. All pulmonary function tests were performed within 3 months before surgery. After selection based on diagnosis criteria, all lung tissue samples used for the study were checked histologically by using the following exclusion criteria: (1) presence of tumour, (2) respiratory tract infection.

The lungs taken from donor controls showed a normal architecture with few intra-alveolar macrophages and oedema. The protocol was approved by the local research and independent ethics committee of the University General Consortium Hospital of Valencia (CEI CHGUV/052016). Informed written consent was obtained from each participant. Clinical data are provided in Table 1.

### 4.2. Culture of A549 and MRC5 Cells

The bronchoalveolar A549 cell line has been used to model ATII behaviour and the MRC5 lung fibroblast cell line has been used to model fibroblast behaviour. Both cells lines were purchased from American Type Culture Collection (Rockville, MD, USA), which authenticates their lines via short tandem repeat profiling. They were cultured in 10% FCS supplemented Roswell Park Memorial Institute (RPMI) 1640 medium at 37 °C in a humidified atmosphere of 5% CO2 in air, as outlined [74]. Cells at 60–70% confluence were serum-deprived by incubation for 12–18 h in RPMI 1640 medium containing 0.1% (*v/v*) foetal bovine serum prior to stimulation with TGFβ1 or other agents.

For in vitro studies, A549 or MRC5 cells were stimulated with recombinant TGF-β1 (Sigma Aldrich; catalogue no. T7039) for the indicated times and concentrations. TGFβ1 has demonstrated to induce cell phenotypic changes such as epithelial to mesenchymal transition at the indicated concentrations [75].

### 4.3. SiRNA Transfection of A549 and MRC5 Cells

Small interfering RNA (siRNA), including the scrambled siRNA control, was purchased from Ambion (Huntingdon, Cambridge, UK; catalogue no. 4390843). MUC16 gene-targeted siRNA (identification no. IDS41226, catalogue no. 4392420) was designed by Ambion. Cells were transfected with siRNA (50 nM) in serum and antibiotic-free medium. After 6 h, the medium was aspirated and replaced with medium containing serum for a further 42 h before cell stimulation. The transfection reagent used was lipofectamine-2000 (Invitrogen, Paisley, UK; catalogue no. 11668-027) at a final concentration of 2 µg/mL.

### 4.4. Western Blotting Analysis

Western blotting analysis was used to detect changes in A549 and MRC5 cell protein expression. Cells were scraped from a confluent 25 cm^2^ flask and lysed on ice with a lysis buffer comprising a complete inhibitor cocktail plus 1 mM ethylenediaminetetraacectic acid (Roche Diagnostics Ltd., West Sussex, UK) with 20 mM Tris base, 0.9% NaCl, 0.1% Triton X-100, 1 mM dithiothreitol, and 1 mg/mL pepstatin A. The Bio-Rad assay (Bio-Rad Laboratories Ltd., Herts, UK) was used according to the manufacturer’s instructions to quantify the level of protein in each sample to ensure equal protein loading. Sodium dodecyl sulphate polyacrylamide gel electrophoresis was used to separate the proteins according to their molecular weight. Briefly, 15 µg of proteins (denatured) along with a molecular weight protein marker (Bio-Rad Kaleidoscope marker; Bio-Rad Laboratories) were loaded onto an acrylamide gel consisting of a 5% acrylamide stacking gel stacked on top of a 10% acrylamide resolving gel and run through the gel by application of 100 V for 1 h. Proteins were transferred from the gel to a polyvinylidene difluoride membrane using a wet-blotting method. The membrane was blocked with 5% bovine serum albumin (BSA) in PBS containing 0.1% Tween20 (PBS-T), probed with the following antibodies: MUC16 (1:1000) antibody (200KDa, monoclonal antibody; SantaCruz Biotechnology, Dallas, USA; catalogue no sc 365002) collagen type I (1:1000) antibody (139 KDa, polyclonal antibody; Calbiochem Darmstadt, Germany; catalogue no. 234167), phospho(p)-SMAD3 (1:1000) antibody (50 KDa, monoclonal antibody; Millipore, Madrid, Spain; catalogue no. PS1023), total anti-β-catenin antibody (92 KDa, polyclonal antibody; Cell Signalling; catalogue no. 9562S) and normalised to total anti-human β-actin (1:1000) antibody (42 KDa, monoclonal antibody, catalogue no. A1978; Sigma) and total anti-Smad3 (1:1000) antibody (48 KDa, polyclonal antibody; Calbiochem, Madrid, Spain; catalogue no. 566414). The enhanced chemiluminescence method of protein detection using enhanced chemiluminescence reagents (ECL Plus; Amersham GE Healthcare, Buckinghamshire, UK) was used to detect labelled proteins. Densitometry of films was performed using the Image J 1.42q software (available at http://rsb.info.nih.gov/ij/, USA). Results of target protein expression are expressed as the percentage of the densitometry of the endogenous controls β-actin or total SMAD3, as appropriate.

### 4.5. Real-Time RT-PCR

Total RNA was isolated using TriPure^®^ Isolation Reagent (Roche, Indianapolis, IN, USA). The integrity of the extracted RNA was confirmed with Bioanalyzer (Agilent, Palo Alto, CA, USA). Reverse transcription was performed in 300 ng of total RNA with a TaqMan reverse transcription reagents kit (Applied Biosystems, Perkin-Elmer Corporation, CA, USA). cDNA was amplified with specific primers and probes predesigned by Applied Biosystems for MUC16 (Hs01065189_m1), α-SMA (Hs00559403_m1), α_1_(I)-collagen (collagen type I; Hs00164004_m1), SNAIL (Hs00195591_m1) and SLUG (Hs00161904_m1) in a 7900HT Fast Real-Time PCR System (Applied Biosystems) using Universal Master Mix (Applied Biosystems). Expression of the target gene was expressed as the fold increase or decrease relative to the expression of β-actin as an endogenous control (Applied Biosystems; Hs01060665). The mean value of the replicates for each sample was calculated and expressed as the cycle threshold (Ct). The level of gene expression was then calculated as the difference (ΔCt) between the Ct value of the target gene and the Ct value of β-actin. The fold changes in the target gene mRNA levels were designated 2^-^^Δ^^Ct^.

### 4.6. Proliferation

Fibroblast proliferation was measured by colorimetric immunoassay based on BrdU incorporation during DNA synthesis using a cell proliferation enzyme-linked immunosorbent assay BrdU kit (Roche, Mannheim, Germany) according to the manufacturer’s protocol. Cells were seeded at a density of 3 × 10^3^ cells/well on 96-well plates and incubated for 24 h. TGF-β1 stimulation was incubated for 48 h. The 490 nm absorbance was quantified using a microplate spectrophotometer (Victor 1420 Multilabel Counter, PerkinElmer). Proliferation data refer to the absorbance values of BrdU-labeled cellular DNA content per well.

### 4.7. Immunoprecipitation

Equal amounts of protein (200 µg) from total protein extracts were incubated with 2 µg of anti-MUC16 antibody. The immune complexes were precipitated with protein G on Sepharose 4B fast flow beads (Sigma Aldrich; catalogue no. P-3296) overnight at 4 °C. After washing three times with NET buffer containing 50 mM Tris-HCl at pH 8.0, 150 mM NaCl, and 0.1% Nonidet P-40, the bound materials were eluted from the immunoprecipitates in reducing SDS-PAGE loading buffer containing 10% SDS, 1 M Tris-HCl at pH 6.8, 50% glycerol, 10% 2-mercaptoethanol, and 2% bromophenol blue at 100 °C for 10 min. Immunoprecipitated protein complexes were assayed by Western blotting, as described above, and probed using p-SMAD3 or anti-MUC16 antibodies, as appropriate.

### 4.8. Histological, Immunohistochemical and Immunofluorescence Studies

For immunohistochemical analysis of human lungs, the tissue was fixed and embedded in paraffin, cut into sections (4–6 µm) and incubated with MUC16 antibody (monoclonal antibody; Abcam, Cambridge, UK; catalogue no. ab10033) overnight at 4 °C. Master Polymer Plus detection system peroxidase (Ref: MAD-000237QKA; master diagnóstica, Granada, Spain) was used for immunostaining development. The non-immune IgG isotype control was used as the negative control and resulted negative for all samples (data not shown).

MUC16 and p-Smad3 was analysed in A549 cells by immunofluorescence. Cells were fixed in paraformaldehyde (4%) for 48 h. Cells were permeabilised (20 mM HEPES at pH 7.6, 300 mM sucrose, 50 mM NaCl, 3 mM MgCl_2_, 0.5% Triton X-100), blocked (10% goat serum in PBS), and incubated with the primary antibodies (MUC16 antibody (monoclonal antibody; Abcam, Cambridge, UK; catalogue no. ab10033) and phospho(p)-SMAD3 antibody (monoclonal antibody; Millipore, Madrid, Spain; catalogue no. PS1023) overnight at 4°C, followed by secondary antibody anti-mouse/rabbit rhodamine/FITC- (1:100, Molecular Probes). Colocalisation of MUC16/ p-Smad3 was performed using a confocal spectral Leica TCS SP2 microscope with 1000 × magnification and 3 × zoom. Red (HeNe 543 nm), green (HeNe 488 nm), and blue (Ar 351 nm, 364 nm) lasers were used. Colocalisation studies were performed using the Leica confocal software v2.61. The cell images with colocalised points of the two laser canals were transformed into a white colour.

### 4.9. SBE Assay in siRNA Transfected Cells

The SBE Reporter kit (Cat#: 60654, BPSBioscience) was used for monitoring the activity of TGFβ/SMAD signalling pathway in A549 cells. One day before transfection, cells were seeded at a density of 2 × 10^4^ cells per well in 200 µL of 10% FBS medium (antibiotic-free), so that cells were 70% confluent at the time of siRNA transfection. The plate was incubated at 37 °C in a CO_2_ incubator. The next day, siRNAs 50 nM were transfected into cells using lipofectamine 2 µg/mL. Cells were incubated at 37 °C for 24 h. After 24 h of siRNA transfection, 1 µg of SBE luciferase reporter vector + constitutively expressing renilla luciferase vector diluted in 22.5 µL of Opti-MEM I medium (antibiotic-free) was prepared. The control transfection was the non-inducible luciferase vector + constitutively expressing renilla luciferase vector. Complexes were incubated for 5 min at room temperature. Diluted DNA was combined with diluted Lipofectamine 2000 and incubated for 25 min at room temperature. Then, 45 µL of complexes were added to each well containing cells and medium, and cells were incubated at 37 °C in a 5% CO_2_ incubator for 24 h. After 24 h, the medium was changed to 0.5% FBS fresh medium and TGF-β1 (final concentration 10 ng/mL) was added to the wells. After 18 h of stimulation, luciferase activity was evaluated by dual-luciferase reporter assay system (Promega, catalogue no. E1910) following manufacturer’s protocol. To obtain the normalised luciferase activity of the SBE reporter, the background was subtracted, and the ratio of firefly luminescence from the SBE reporter to renilla luminescence from the control renilla luciferase vector was represented.

### 4.10. Statistical Analysis

Statistical analysis of results was carried out by parametric (cellular studies) or non-parametric (human studies) analysis, as appropriate. *p* < 0.05 was considered statistically significant. Non-parametric tests were used to compare results from human samples of control patients and IPF patients (Figure 1 in main manuscript). In this case, data were displayed as medians, interquartile range, and minimum and maximum values. When the comparisons concerned only two groups, between-group differences were analysed by the Mann–Whitney test. Results from cellular in vitro experiments were expressed as the mean ± standard error (SE) of n experiments. In this case, statistical analysis was carried out by parametric analysis. Two-group comparisons were analysed using the Student’s *t*-test.

## Figures and Tables

**Table 1:** Clinical characteristics of human lung tissue samples used.

	Control Donor Subjects (*n* = 17)	IPF Patients (*n* = 20)	Mean Difference (CI)	*p*-Value
**Age (yr)**	59 (53.5–62)	61 (55.5–64.5)	2 (−7 to 2)	0.2516
**Sex (Male/Female)**	13/4	15/5		
**Smoking**				
Never smoked/Smokers	3/14	4/16		
Pack-year	25 (20–30)	26 (10.5–32.5)	1 (−8 to 4)	0.4782
**FEV1, pred**	ND	62 (56.5–70.5)		
**FVC, % pred**	ND	55 (51–65.5)		
**DLco, % pred**	ND	34 (25–44)		
**PaO_2_, mmHg**	94 (90.5–95)	51 (45.5–60.5)	41.8 (35 to 46)	<0.001 *
**NAC (y/n)**	0	16/6		
**Pirfenidone (y/n)**	0	6/16		

% pred, % predicted; DLco, diffusion capacity of the lung for carbon monoxide; FEV1, forced expiratory volume in 1 s; FVC, forced vital capacity; ND, not determined; Pack-year, 1 year of smoking 20 cigarettes per day; PaO_2_, arterial blood oxygen tension. N-acetyl-l-cysteine (NAC)/pirfenidone refers to patients who received this treatment at the time of pulmonary sample. * compared with control donors. None of the patients received nintedanib. Data are presented as medians [interquartile rang].

## Data Availability

Not applicable.

## Supplementary Materials

The following are available online at https://www.mdpi.com/article/10.3390/ijms22126502/s1.
