# Supplementary material for: MUC16 Is Overexpressed in Idiopathic Pulmonary Fibrosis and Induces Fibrotic Responses Mediated by Transforming Growth Factor-β1 Canonical Pathway
Source: Int J Mol Sci. 2021 Jun 17;22(12):6502. doi: 10.3390/ijms22126502 (PMC8235375; doi:10.3390/ijms22126502)

Figure 1

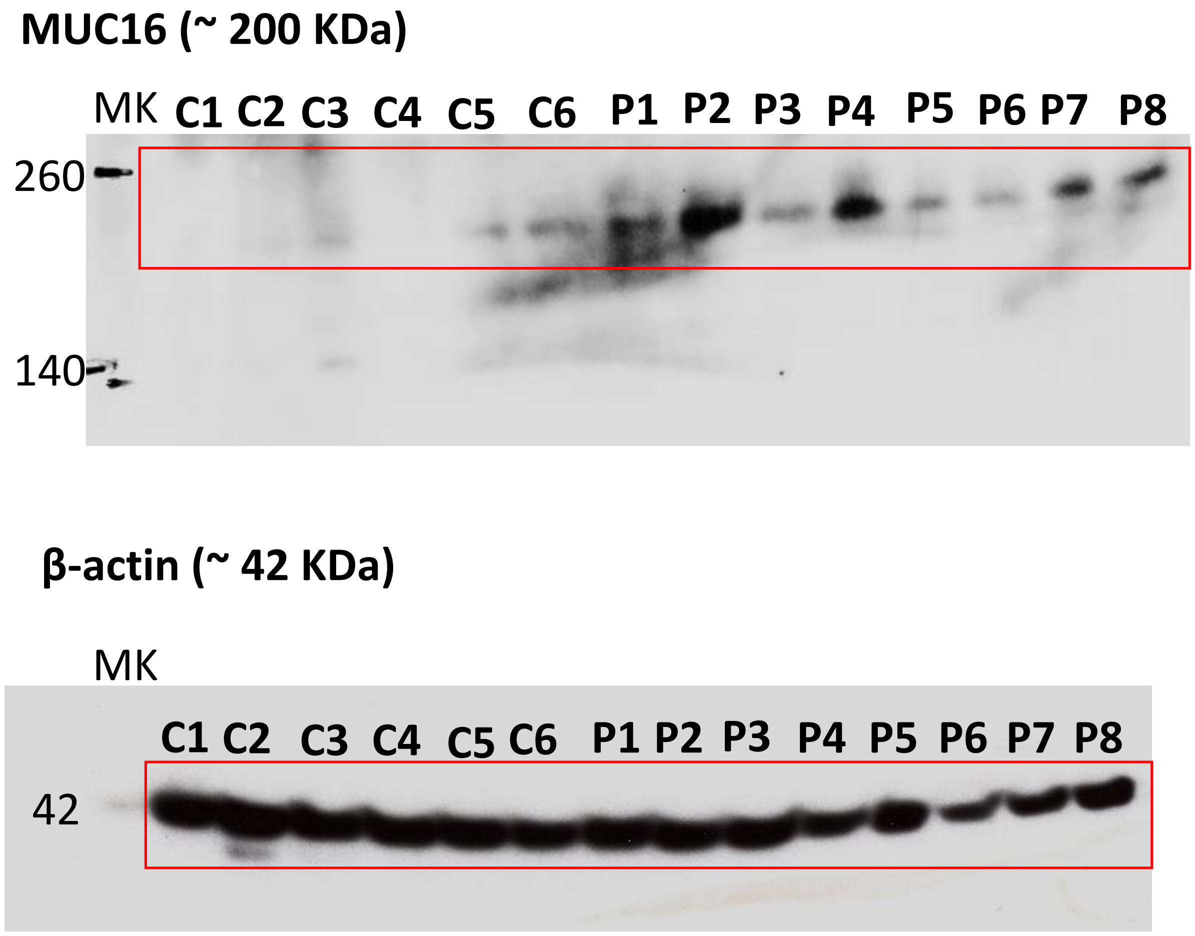

MUC16 (~ 200 KDa)
MK C1 C2 C3 C4 C5 C6 P1 P2 P3 P4 P5 P6 P7 P8
260
140
β-actin (~ 42 KDa)
MK
C1 C2 C3 C4 C5 C6 P1 P2 P3 P4 P5 P6 P7 P8
42

Figure 2

Col type I (139 KDa)

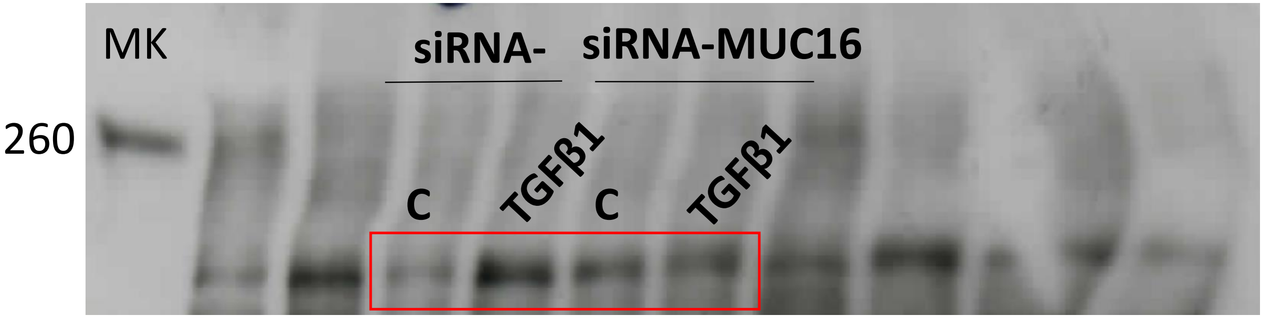

β-actin (~ 42 KDa)

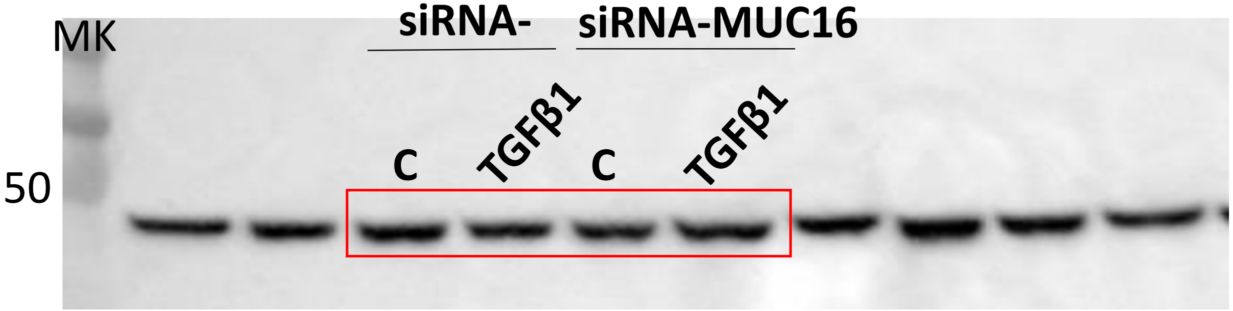

Figure 3

Col type I (139 KDa)

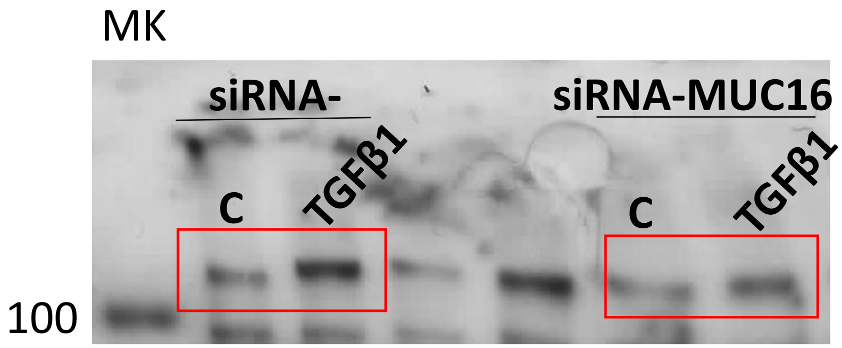

β-actin (~ 42 KDa)

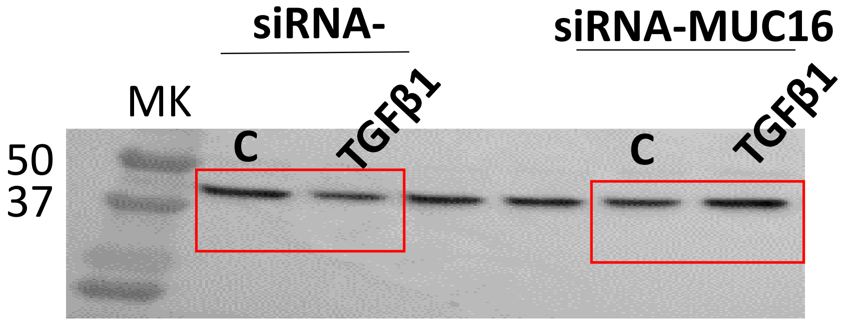

Figure 5

A549

P-Smad3 (~50 KDa)

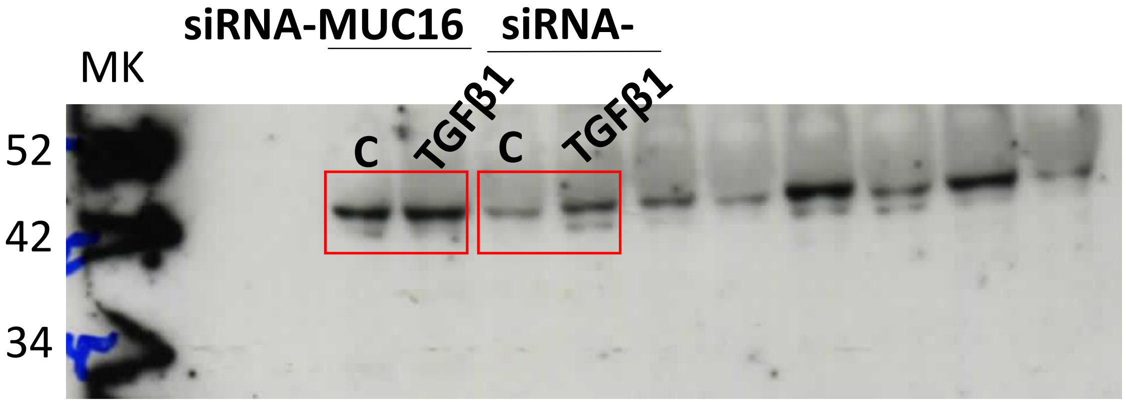

β-catenin (~ 96 KDa)

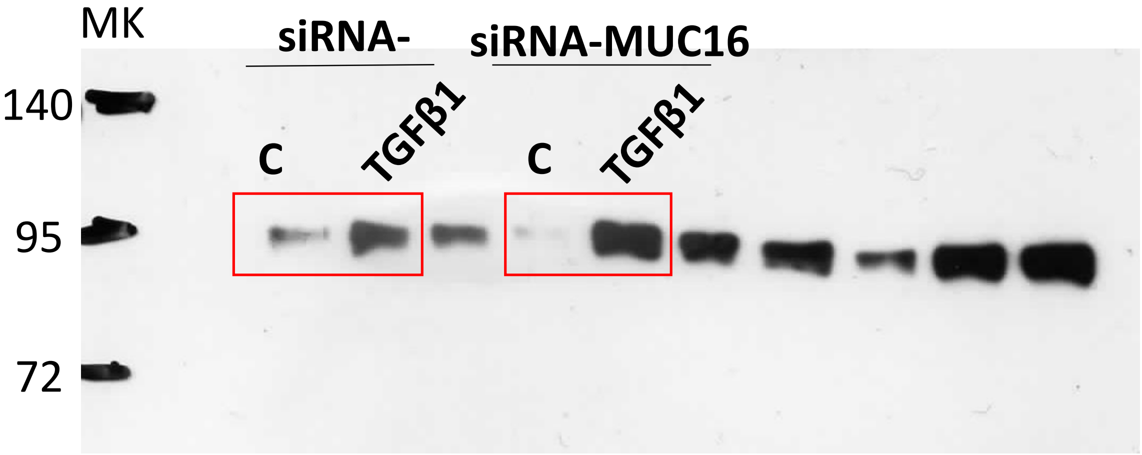

Figure 5

A549

Smad3 (~50 KDa)

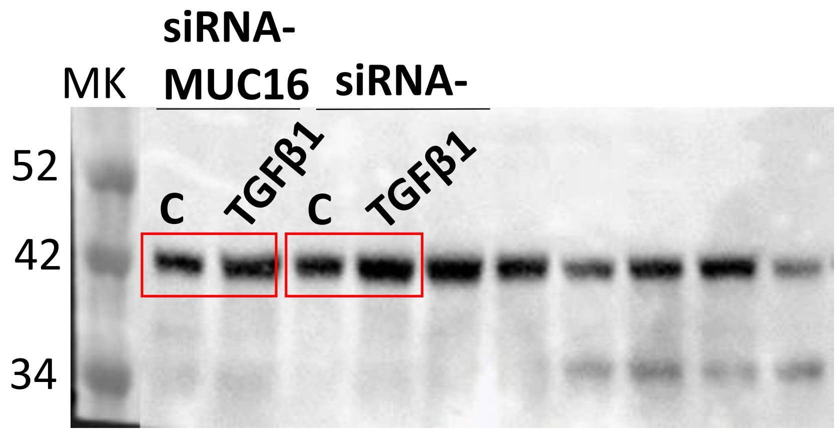

β-actin (~ 42 KDa)

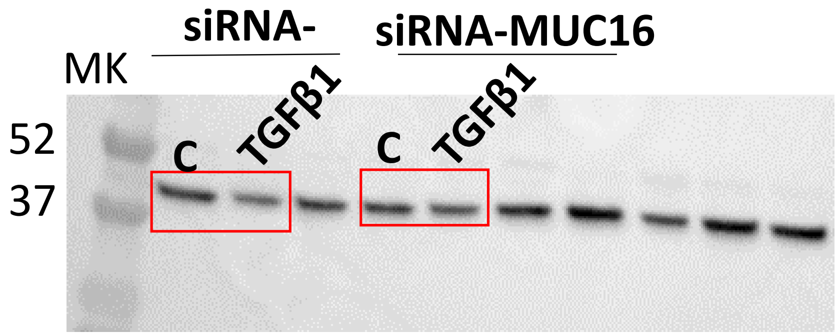

Figure 5

MRC5

P-Smad3 (~50 KDa)

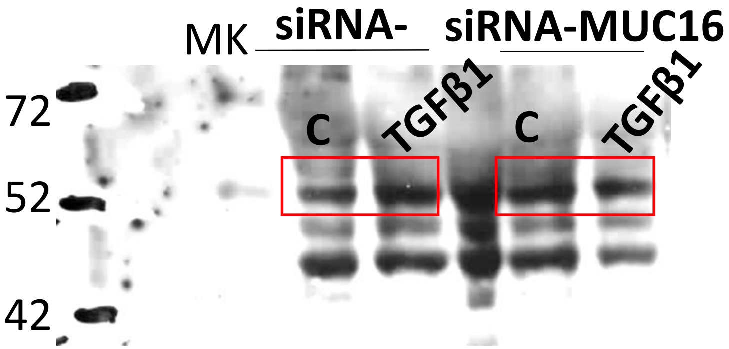

β-catenin (~ 96 KDa)

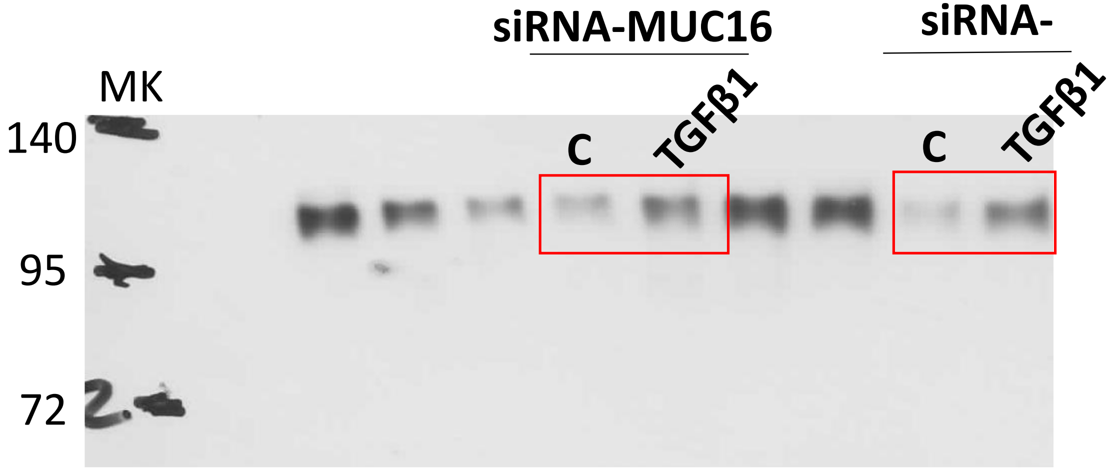

Figure 5

MRC5

Smad3 (~50 KDa)

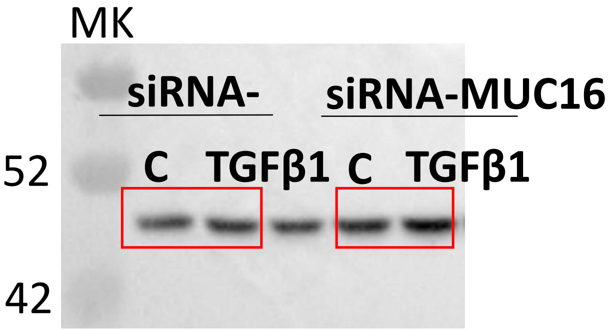

β-actin (~ 42 KDa)

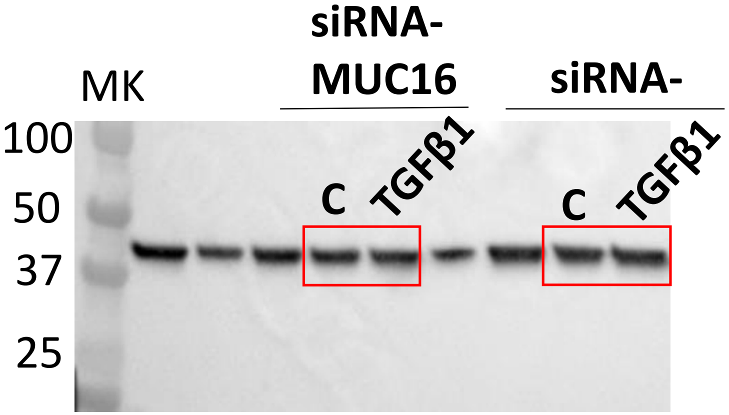

Figure 5

IP MUC16; A549 IP MUC16; MRC5

P-Smad3 (~50 KDa)

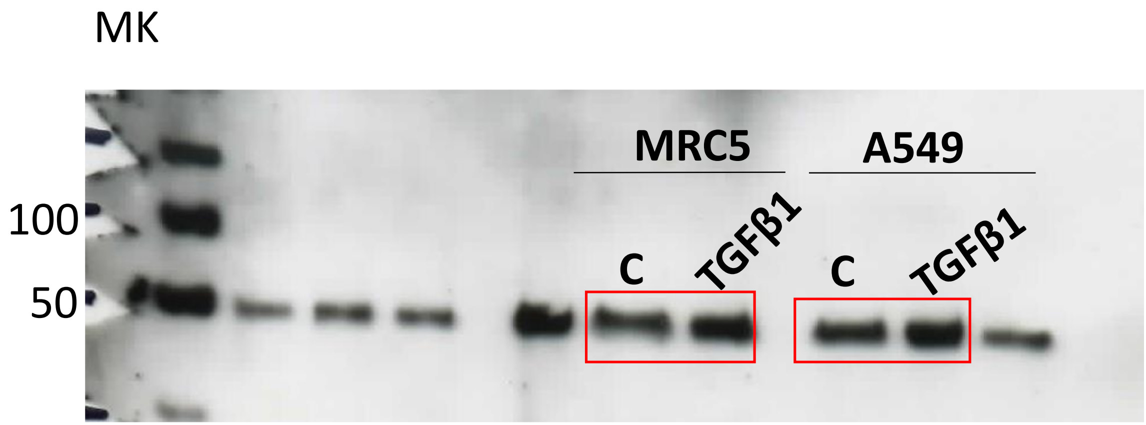

IP MUC16; A549 IP MUC16; MRC5

MUC16 (~200 KDa)

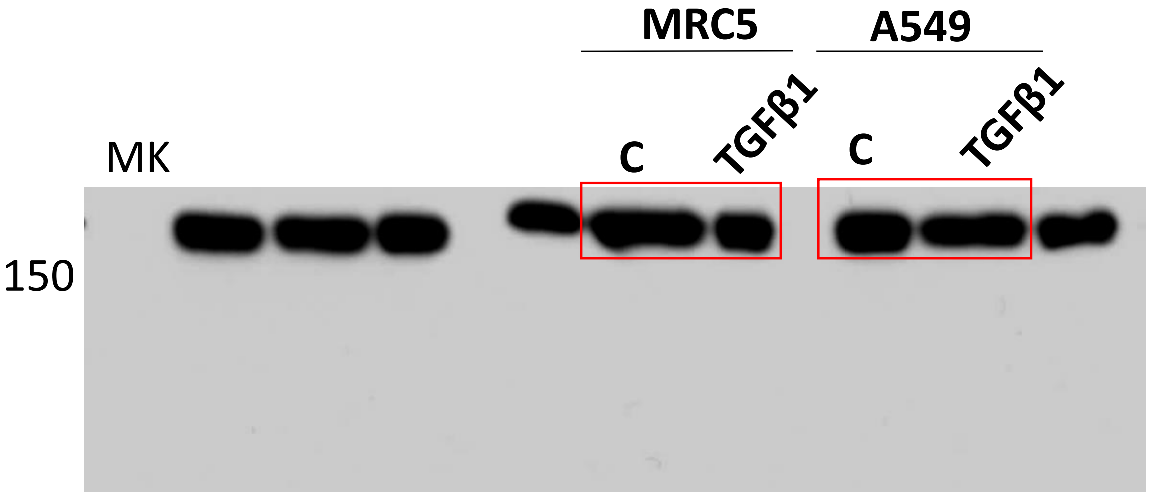

Supplement: Supplementary file 1 [file ijms-22-06502-s001.zip › original blots.pdf]
