# Supplementary material for: MUC16 Is Overexpressed in Idiopathic Pulmonary Fibrosis and Induces Fibrotic Responses Mediated by Transforming Growth Factor-β1 Canonical Pathway
Source: Int J Mol Sci. 2021 Jun 17;22(12):6502. doi: 10.3390/ijms22126502 (PMC8235375; doi:10.3390/ijms22126502)
Supplement: Supplementary file 1 [file ijms-22-06502-s001.zip › supplementary.pdf]

TITLE

MUC16 is overexpressed in idiopathic pulmonary fibrosis and induces fibrotic responses mediated by transforming growth factor-β1 canonical pathway

Authors

Beatriz Ballester [1,2*] PhD; Javier Milara [2,3,4*] PharmD, PhD; Paula Montero [4]; Prof. Julio Cortijo[2,4,5] PharmD, PhD.

Author Affiliations

[1] Comprehensive Pneumology Center (CPC), Helmholtz Zentrum München, München, Germany.
[2]CIBERES, Health Institute Carlos III, Valencia, Spain
[3]Pharmacy Unit, General University Hospital, Valencia, Spain
[4]Department of Pharmacology, Faculty of Medicine, University of Valencia, Spain
[5]Research and teaching Unit, University General Hospital Consortium, Valencia, Spain
*Both authors contributed equally to this work

Corresponding author:

Beatriz Ballester, PhD; Comprehensive Pneumology Center (CPC), Helmholtz Zentrum München. Max-Lebsche-Platz 31, 81377, München, Germany; +34 605148470; E-mail: beatriz.ballester@helmholtz-muenchen.de

Javier Milara, Pharmacy unit, University General Hospital Consortium of Valencia, Spain. Phone: +34 963864631: E-mail: xmilara@hotmail.com

Key words: Idiopathic pulmonary fibrosis; MUC16; transforming growth factor beta

Supplementary Figures

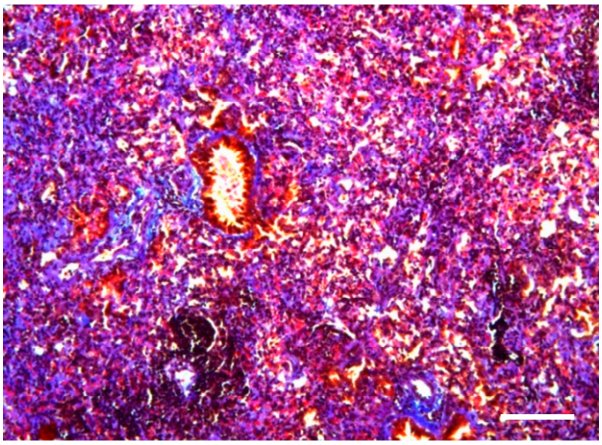

Supplementary Figure 1

Supplementary Figure 1. Masson's trichrome representative staining on IPF lung sections (scale bar: 100 µm).
