# Supplementary figures and images for: MUC16 Is Overexpressed in Idiopathic Pulmonary Fibrosis and Induces Fibrotic Responses Mediated by Transforming Growth Factor-β1 Canonical Pathway
Source: Int J Mol Sci. 2021 Jun 17;22(12):6502. doi: 10.3390/ijms22126502 (PMC8235375; doi:10.3390/ijms22126502)

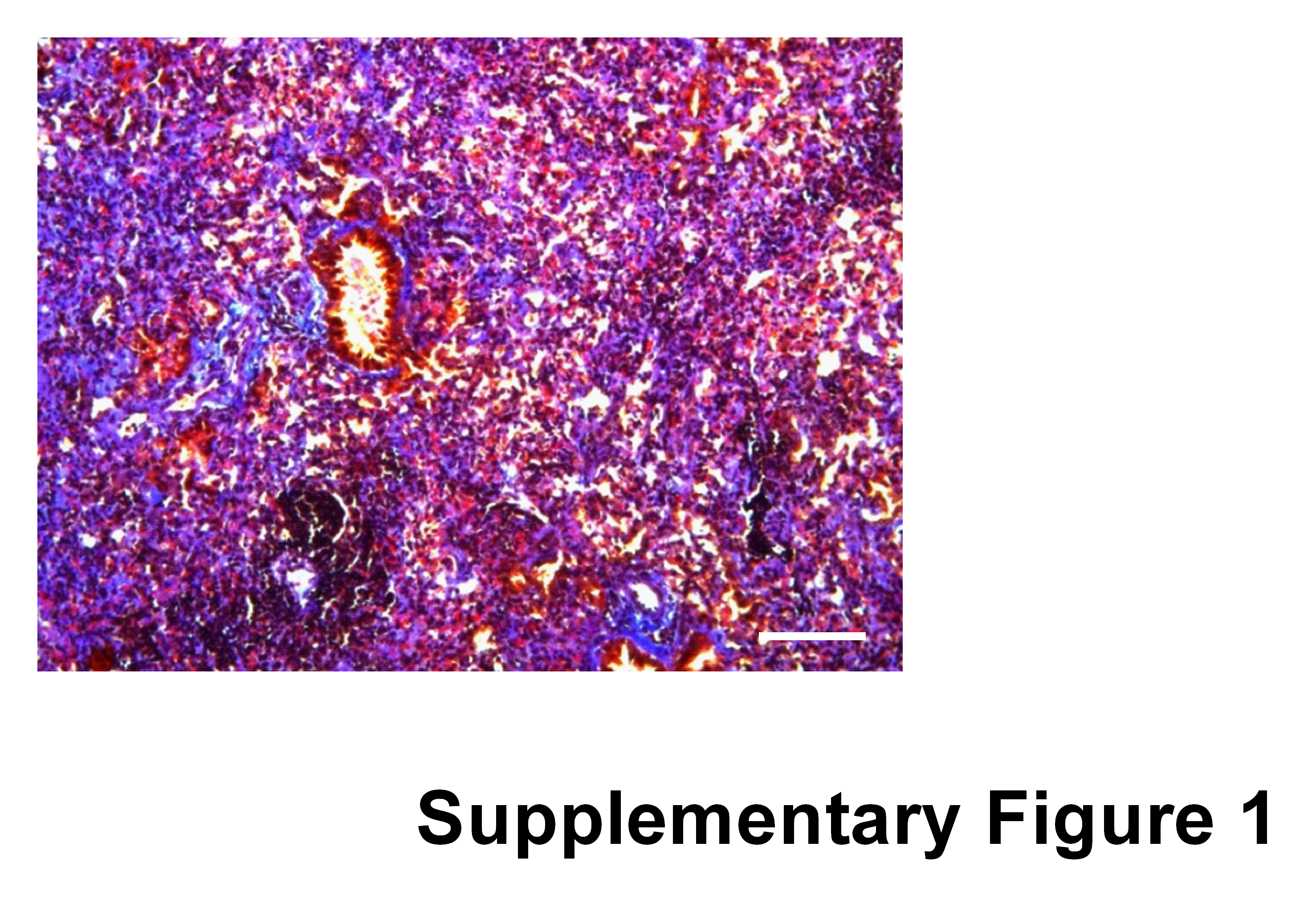

Supplement: Supplementary file 1 [file ijms-22-06502-s001.zip › supplementary Figure 1.tif]
